# Desiccation tolerance in the Antarctic moss *Sanionia uncinata*

**DOI:** 10.1186/s40659-019-0251-6

**Published:** 2019-08-21

**Authors:** Marisol Pizarro, Rodrigo A. Contreras, Hans Köhler, Gustavo E. Zúñiga

**Affiliations:** Laboratorio de Fisiología y Biotecnología Vegetal, Departamento de Biología, Facultad de Química y Biología, and CEDENNA, Universidad de Santiago de Chile, Av. Libertador Bernardo O’Higgins 3363, Estación Central, Santiago Chile

**Keywords:** Antarctic mosses, ABA, Water stress, Desiccation tolerance, Maritime Antarctic

## Abstract

**Background:**

One of the most extreme environments on our planet is the Maritime Antarctic territory, due to its low-water availability, which restricts the development of plants. *Sanionia uncinata* Hedw. (Amblystegiaceae), the main colonizer of the Maritime Antarctic, has effective mechanisms to tolerate this environment. It has been described that the tolerance to desiccation is mediated by the hormone abscisic acid (ABA), antioxidants systems, accumulation of compatible solutes and proteins of the late embryogenesis abundant (LEA). However, to date, these mechanisms have not been described in *S. uncinata*. Therefore, in this work, we postulate that the tolerance to desiccation in the Antarctic moss *S. uncinata* is mediated by the accumulation of ABA, the osmolytes proline and glycine betaine, and dehydrins (an LEA class 11 proteins). To demonstrate our hypothesis, *S. uncinata* was subjected to desiccation for 24 h (loss in 95% of water content), and the effects on its physiological, photosynthetic, antioxidant and biochemical parameters were determined.

**Results:**

Our results showed an accumulation of ABA in response to water loss, and the activation of protective responses that involves an increment in levels of proline and glycine betaine, an increment in the activity of antioxidant enzymes such as SOD, CAT, APX and POD, and the accumulation of dehydrins proteins.

**Conclusion:**

The results showed, suggest that *S. uncinata* is a  desiccation-tolerant moss, property mediated by high cellular plasticity regulated by ABA.

## Background

Water availability is a limiting factor for any lifeform, but some plants have developed a mechanism that allows them to avoid or deal with water scarcity, especially those species that evolve during desiccation events [1, 2]. Desiccation tolerance is defined as the ability to survive the decrease of the intracellular water content above 90 percent without irreversible damage [3, 4]. One of the largest groups of plants that can survive low water availability is bryophytes. This taxon belongs to the first lineage of land plants and most of them are desiccation tolerant [5]. Bryophytes are subdivided into mosses, liverworts, and hornworts, they have evolutive traits that give them an advantage over other species to colonize places with low water availability. These characteristics include; small size, radial growth, cell by cell water transport, external water transport, the presence of rhizoids to attach to rocks, wood and other surfaces [5].

*Sanionia uncinata* (Amblystegiaceae) is one of the most abundant mosses colonizing maritime Antarctica; in this region, plants can experience very harsh environmental conditions such as sub-zero temperatures, poor soils, strong winds, fluctuations in UVB and PAR radiation and water deficit [6].

In general, plants have the capacity to respond against water depletion using mechanisms that involve the accumulation of compatible solutes like sugars and amino acids. These serve as osmoprotectant compounds stabilizing enzymes and membranes [1], accumulation of LEA (late embryogenesis proteins) proteins, specially dehydrins have protective properties described in vitro when interacts with DNA, proteins, and membranes, also increases antioxidants molecules to control de oxidative burst and the potential damage by reactive oxygen species (ROS), these molecules can be enzymatic or non-enzymatic [7]. The enzymatic response involves the activity of SOD, a metalloenzyme that acts dismutating the superoxide ion (O_2_^−^) into hydrogen peroxide (H_2_O_2_), this molecule can be transformed into H_2_O by ascorbate peroxidases (APX), catalases (CAT) or other types of peroxidases like POD [7].

Current studies show that many responses to dehydration in plants are regulated by abscisic acid, ABA, this phytohormone participates in stress signaling regulating the stomatal conductance, induces antioxidant enzymes [8], the accumulation of osmolytes [9] and induces late embryogenesis proteins [10]. There are novel studies that show the signaling pathway in model plants but there are no studies that show the participation of this hormone in the desiccation tolerance of Antarctic mosses.

*Sanionia uncinata* is one of the most abundant mosses that colonize Maritime Antarctic, belongs to the Amblystegiaceae family and there are no current studies that explain how these organisms can respond to the Antarctic ambient, especially to the drastic changes in water availability.

## Results

**Fig. 1:**
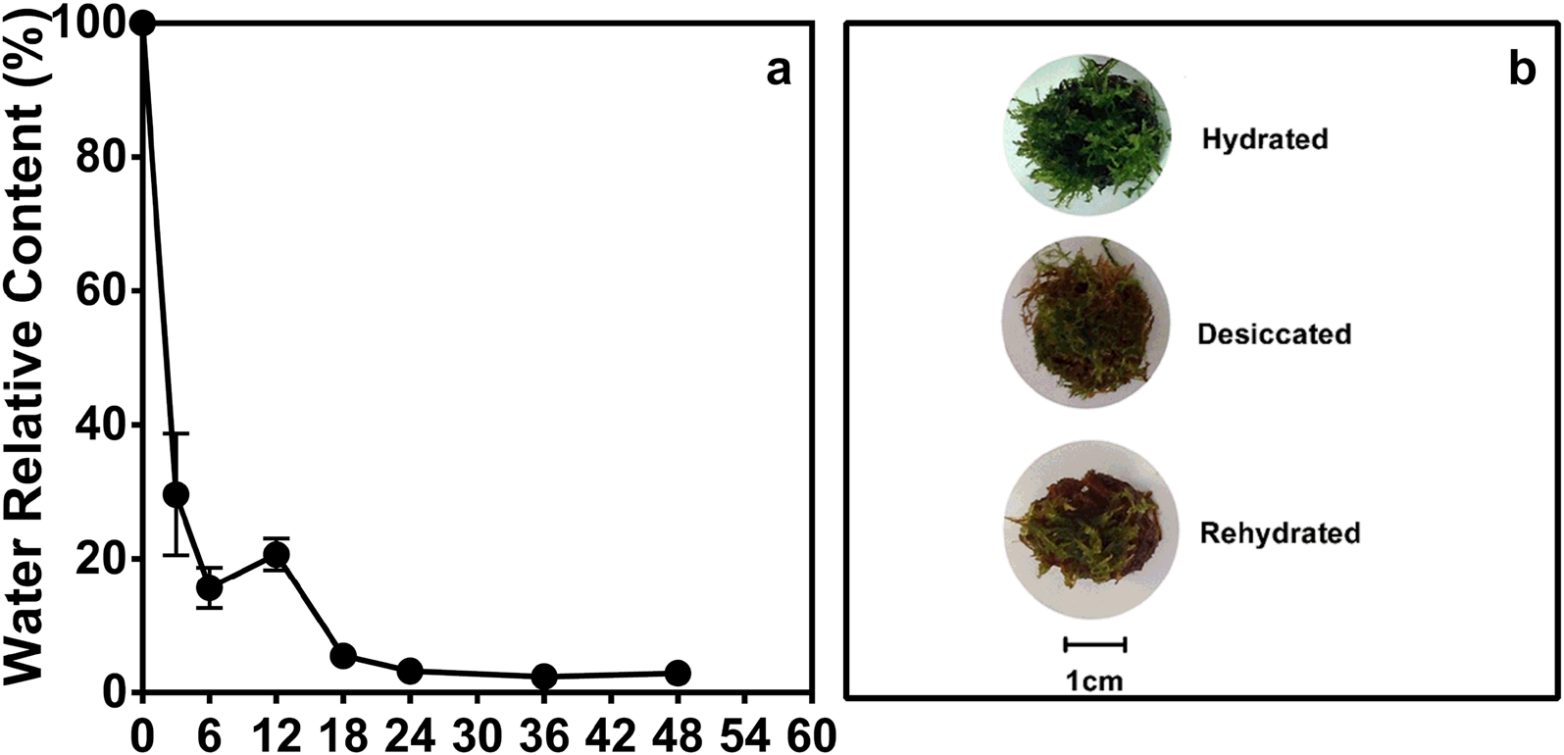
Effect of desiccation in *S. uncinata* plants. Water relative content (**a**), morphology (**b**). The scale bar indicates a length of 1 cm

In the conditions of drying, *S. uncinata* experienced a rapid loss of water during the first 6 h (84% loss) (Fig. 1a). Then, between 6 and 24 h, the loss of water from the moss was slower, reaching a minimum value of water in tissues of 5% after 24 h (Fig. 1a). Under these conditions *S. uncinata* showed a reduction in the volume of phyllids and cauloids, and a morphological change was triggered by desiccation were the photosynthetically active tissue, phyllids, was covered by the brown tissue of cauloids (Fig. 1b). With this result, kinetic desiccation (24 h) -rehydration (36 h) was performed.

**Fig. 2:**
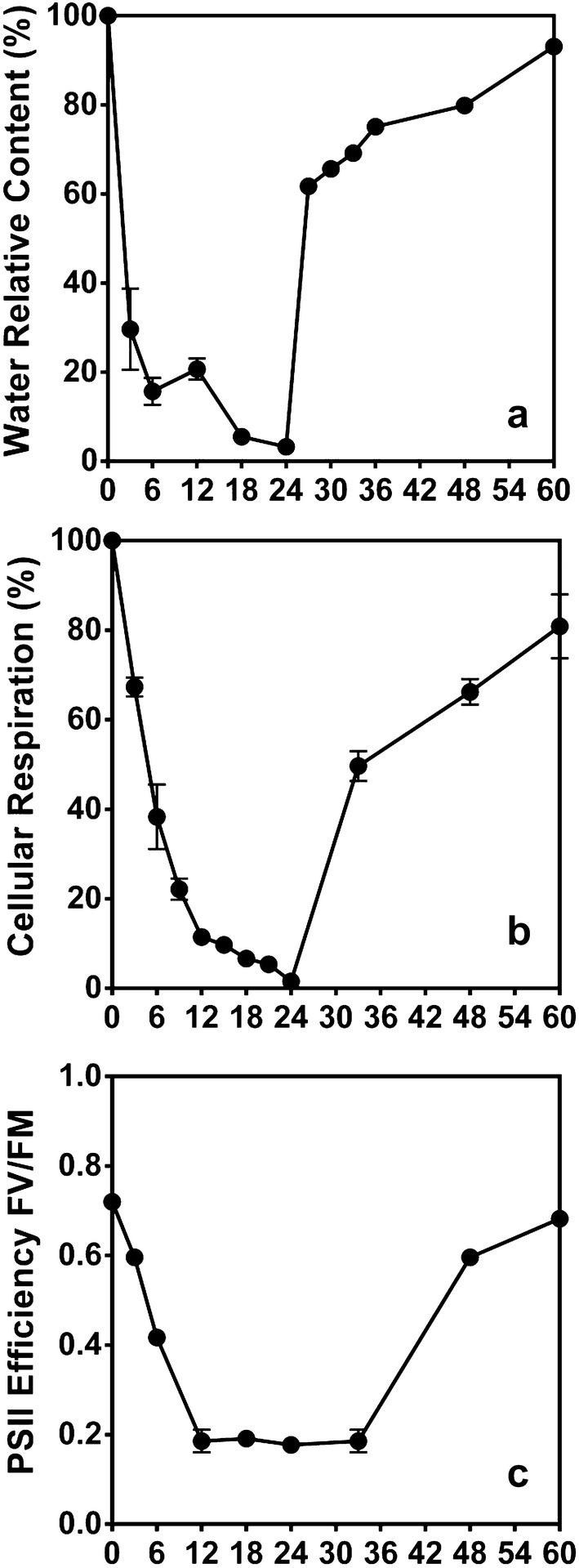
Effect of desiccation on physiological parameters in *S. uncinata* plants. Water content percentage (**a**), cellular respiration percentage (**b**), photosynthetic efficiency (**c**). Each point represent means (± standard error of the mean, N = 3), P < 0.05

**Fig. 3:**
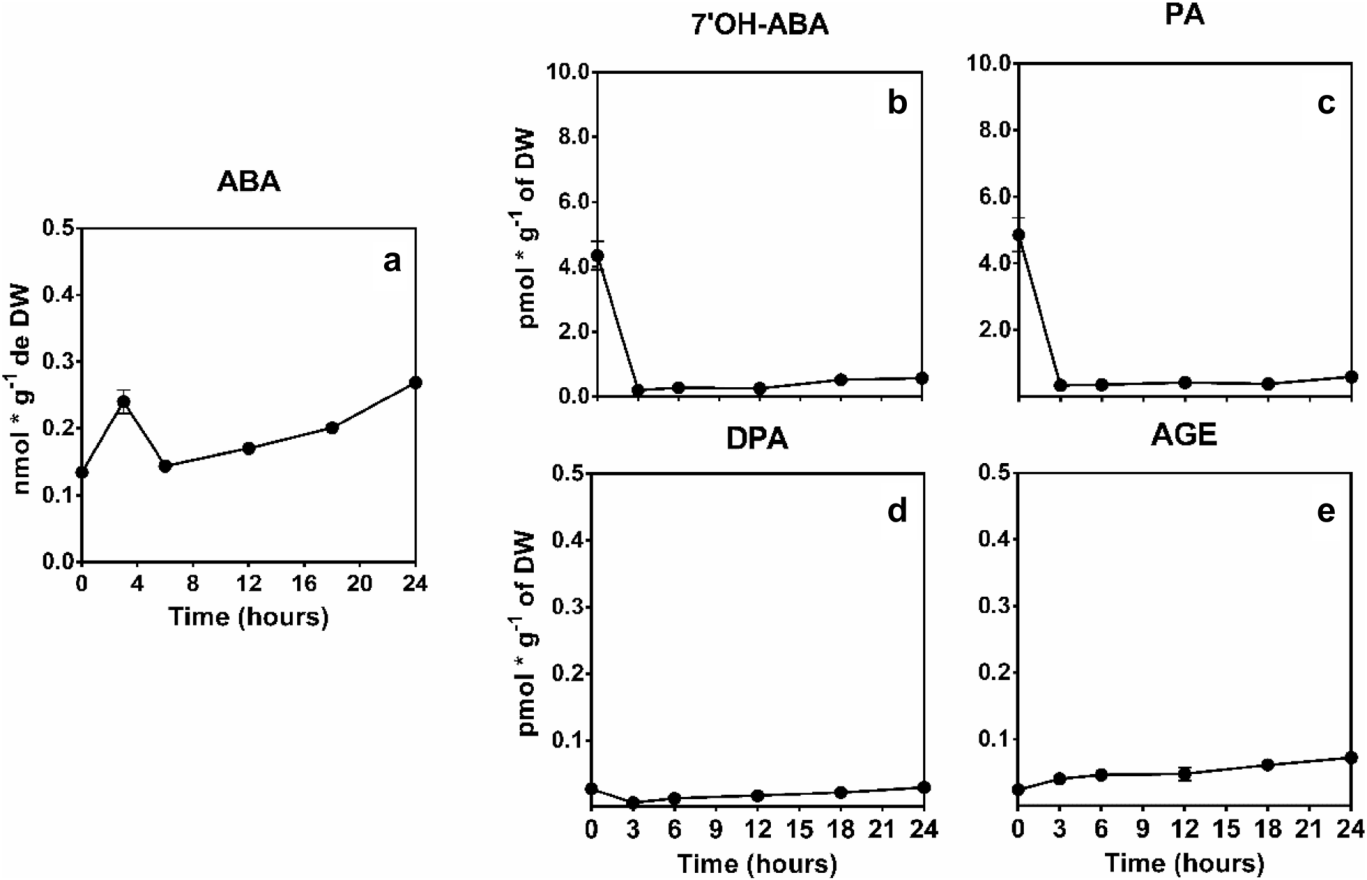
Effect of desiccation in ABA levels and their catabolites. ABA content (**a**), 7′OH-ABA content (**b**), phaseic acid PA content (**c**), dihydrophaseic DPA content (**d**), abscisic acid glucosyl AGE (**e**) ester are shown. Each point represent means (± standard error of the mean, N = 3), P < 0.05

The Antarctic moss shows a 95% reduction of the water content at 24 h of dehydration, but the moss can recover almost 80% of their water content when is rehydrated (Fig. 2a). The physiological responses of the moss show that cellular respiration decrease when *S. uncinata* is desiccated but when the moss is rehydrated increase the respiration levels (Fig. 2b). The photosynthetic efficiency was affected negatively by desiccation at the beginning of desiccation the FV/FM value was 0.628 but at the end the decrease to 0.2 (Fig. 2c). *S. uncinata* submitted to desiccation shows an increase in ABA content, 3.5-fold, also we evaluate the content of ABA catabolites, but there is no increase in their levels (Fig. 3).

**Fig. 4:**
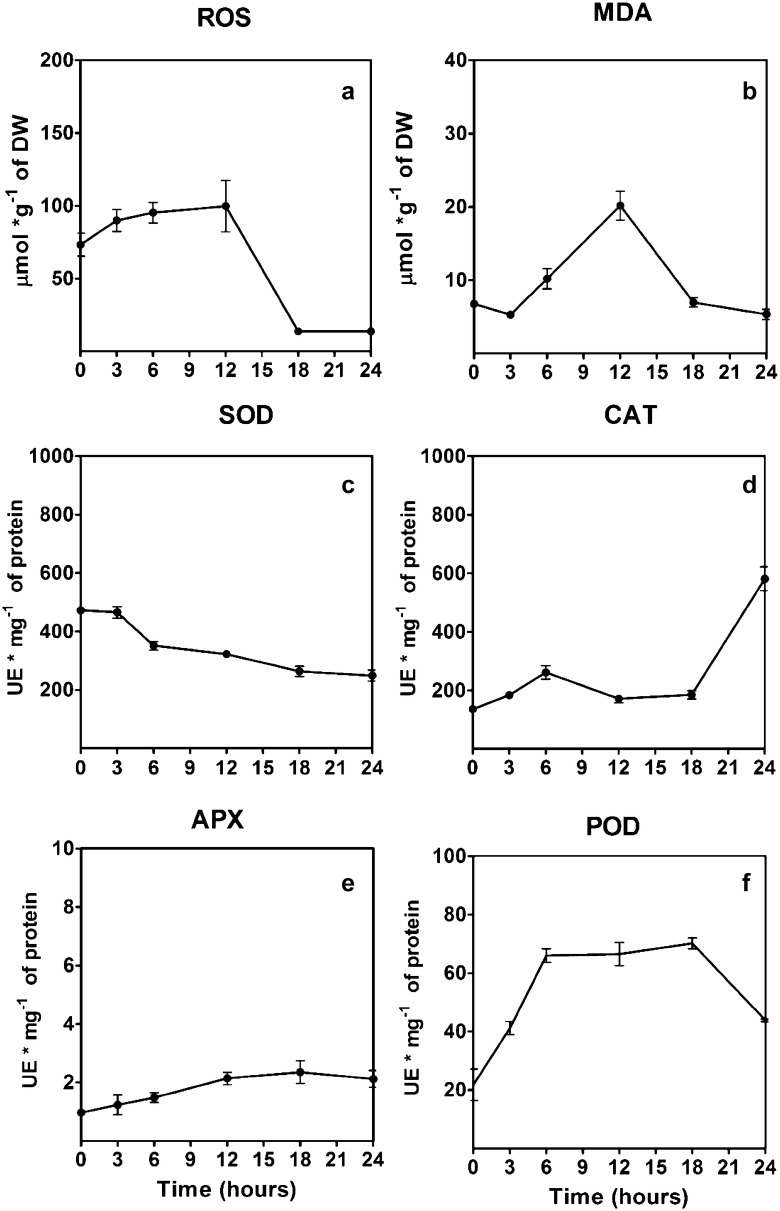
Effect of desiccation in the antioxidant response of *S. uncinata* plants desiccated for 24 h. The content of total ROS levels (**a**), malondialdehyde levels (**b**), superoxide dismutase activity (**c**), catalase activity (**d**) ascorbate peroxidase activity (**e**), the total activity of peroxidases (**f**) is observed. Each point represent means (± standard error of the mean, N = 3), P < 0.05

The homeostasis redox of *S. uncinata* was evaluated, the moss shows an slight increase in the ROS levels when was compared with the beginning of desiccation reaching a peak at the 12 h of treatment, but at the end of the treatment the moss presented a 81% of ROS levels reduction (Fig. 4a), this response is concomitant with an increase in the lipoperoxidation of membrane levels at 12 h of treatment, but at the end of the treatment returns to the basal levels (Fig. 4b), on the other hand, the antioxidant activity of SOD was negatively affected by desiccation decreasing a 47% their activity; the CAT activity increased 327% when the moss was subjected to desiccation, APX increases a 90% and POD a 218% (Fig. 4c, d).

**Fig. 5:**
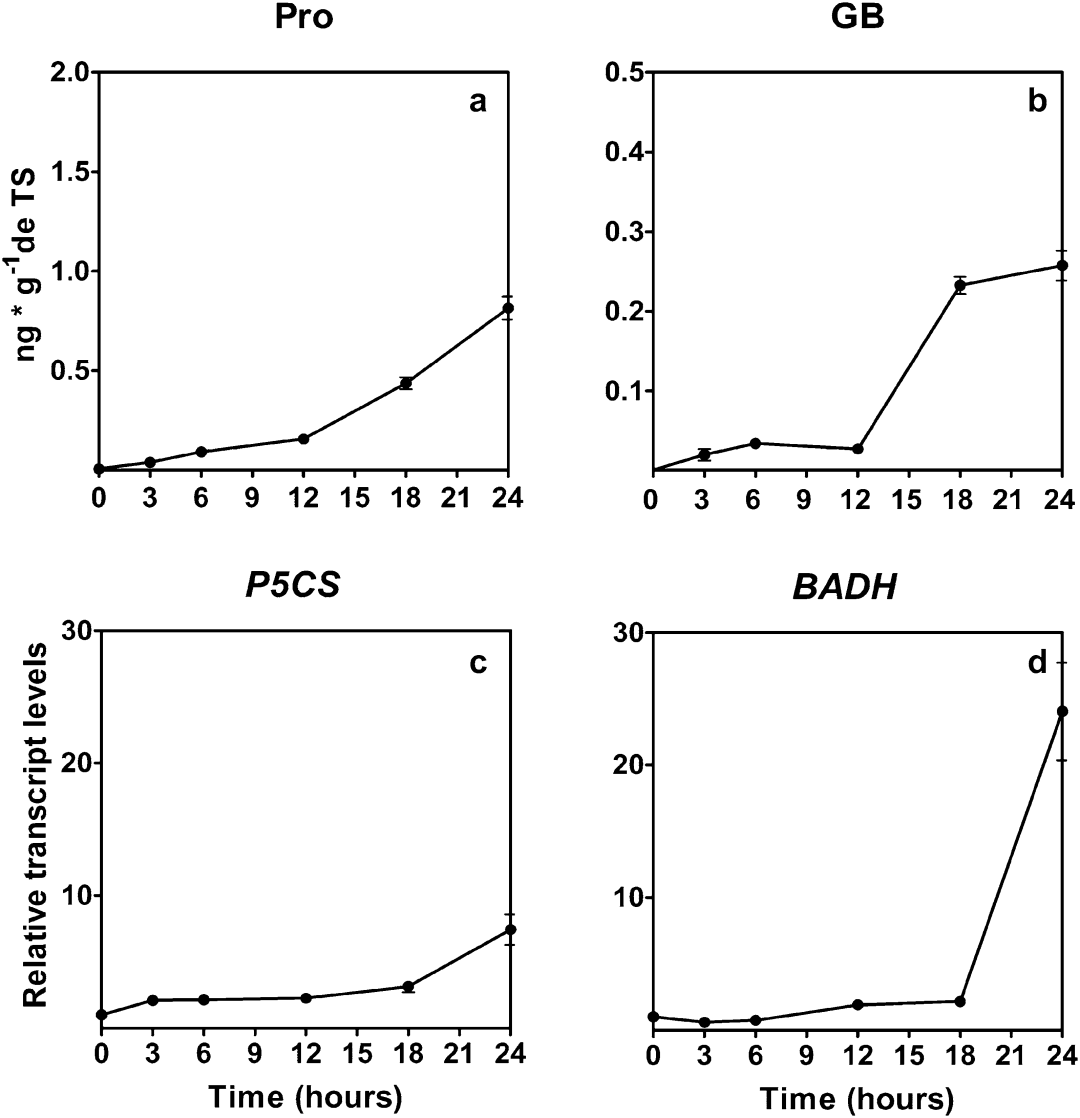
Effect of desiccation in the osmolytes and transcript levels of P5CS and BADH genes in *S. uncinata* plants desiccated for 24 h. The content of proline (**a**), glycine betaine content (**b**), relative transcript levels of P5CS gene (**c**), relative transcript levels of BADH gene (**d**) are shown. Each point represent means (± standard error of the mean, N = 3), P < 0.05

The osmolyte accumulation against desiccation show that *Sanionia* responds increasing the levels of proline 1565-fold and glycine betaine 367-fold (Fig. 5a, b), also the transcripts of genes that codified for the key enzymes in the biosynthesis of proline the pyrroline-5-carboxylate synthase (P5CS) 7.4-fold and the gene betaine-aldehyde dehydrogenase 24-fold (BADH) involved in the glycine betaine biosynthesis increase when the moss is subject to desiccation (Fig. 5c, d).

**Fig. 6:**
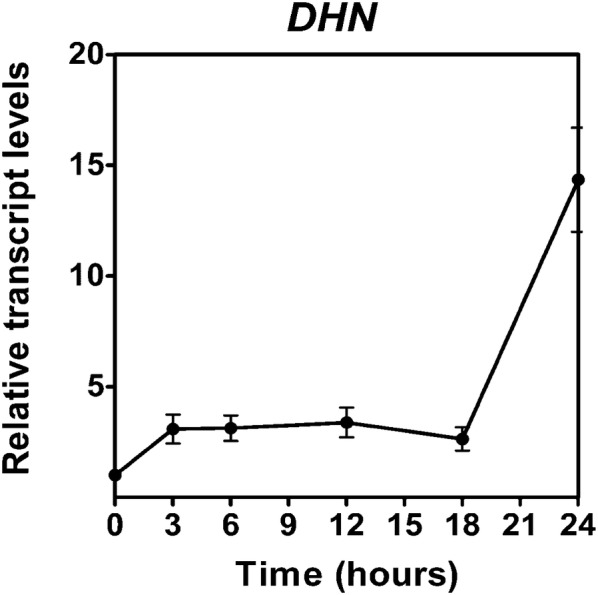
Effect of desiccation in the dehydrins transcript levels of *S. uncinata* plants desiccated for 24 h. Each point represent means (± standard error of the mean, N = 3), P < 0.05

**Fig. 7:**
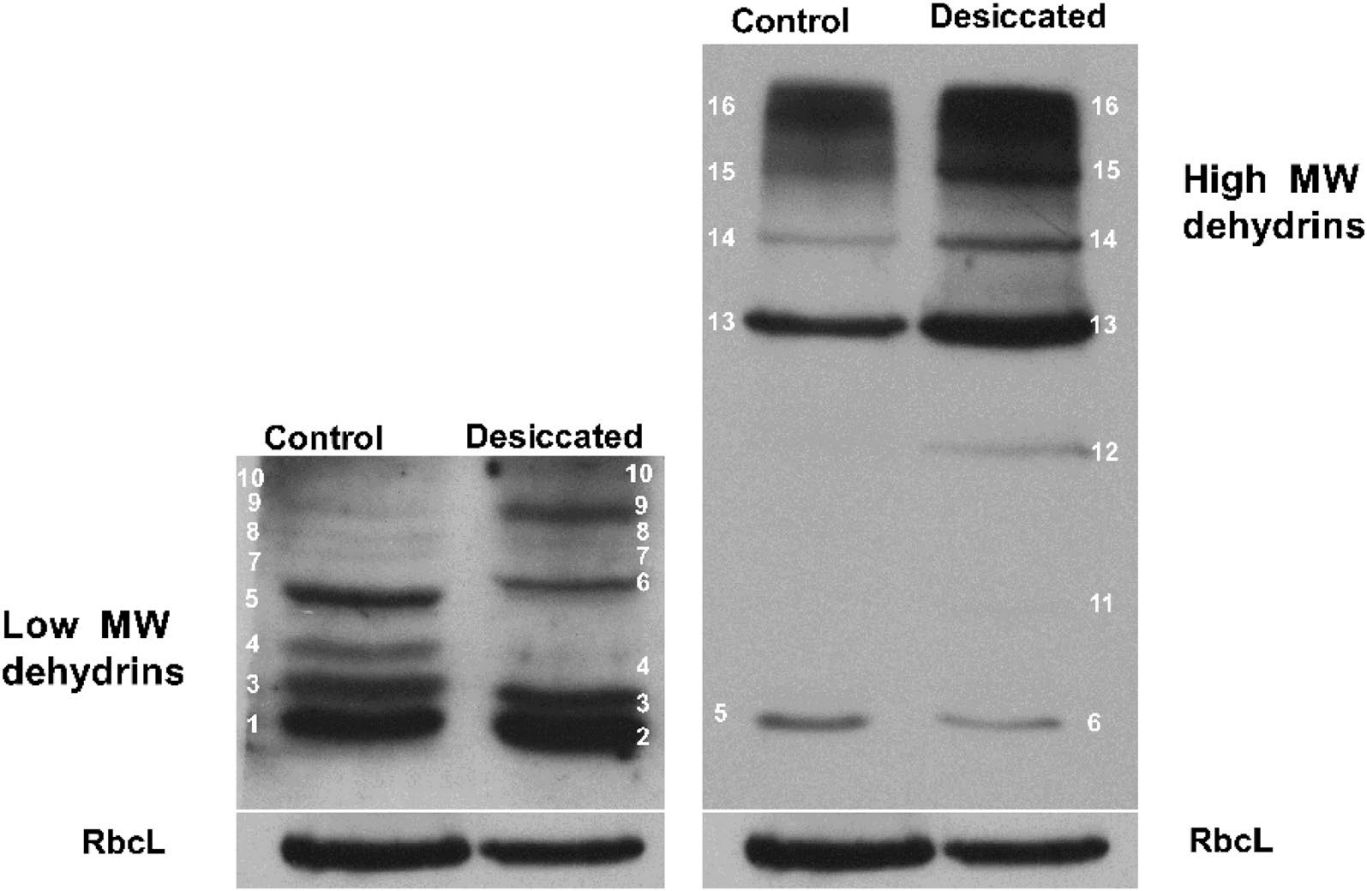
Western blot of the isoforms of dehydrins of *S. uncinata* desiccated for 24 h. (1) 20 kDa, (2) 19 kDa, (3) 21 kDa, (4) 22 kDa, (5) 24 kDa, (6) 25 kDa, (7) 26 kDa, (8) 27 kDa, (9) 28 kDa, (10) 29 kDa, (11) 40 kDa, (12) 50 kDa, (13) 70 kDa, (14) 80 kDa, (15) 100 kDa, (16) 150 kDa, RbcL 55 kDa

The analysis of the transcript levels shows an increase of 14-fold, and the western blot analysis shows that *S. uncinata* (Fig. 6). The western blot analysis shows that the moss present 13 isoform under control conditions (20, 21, 22, 24, 26, 27, 28, 29, 50, 70, 80, 100, 150 kDa) but when is desiccated present 14 isoforms, the 24 kDa isoform is not present and two new forms 25 and 40 kDa are present, also *S. uncinata* experiment an increment in the levels of the 19, 21, 50, 70, 80, 100 and 150 kDa dehydrins (Fig. 7).

## Discussion

The study of the behavior of *S. uncinata* to desiccation allows to determinate which responses are activated. *S. uncinata* shows a fast dehydration rate in 24 h, reaching 5% of water content. The environmental water reduction induces an intracellular water reduction that affects the shape of the moss (Fig. 1) and the solvation shell of cytoskeletal proteins, vacuoles size and reduction on the membrane fluidity [4].

In *S. uncinata* the decrease of water content is related to an increase in ABA levels, these responses present to peaks, the first one is related to the release of the hormone from the ABA conjugates. ABA conjugates are the major reservoir of the inactive hormone; these mechanisms ensure rapid signaling when the molecules are hydrolyzed [11]. The second peak is related to the novo biosynthesis, these processes occur to a minor velocity because it implies transcription of the enzymes genes that are involved in the biosynthetic pathway [12]. ABA it is well known to participate in seed development, stomatal conductance and stress responses in vascular plants, but some studies show that ABA also can be present in non-vascular plants, the pretreatment confers desiccation tolerance to *P. patens* and ABI 3 is the transcription factor that responds to this hormone, when de A, B, C ABI3 genes were deleted, the responsive genes were not upregulated [13]. In *Syntrichia caninervis* they observed upregulation in the AP2-ERF genes during desiccation [14]. Other studies show that ABA treatment in *Atrichum undulatum* increases the desiccation tolerance through the photosynthetic efficiency, increasing F_0_ and the non-photochemical quenching of the moss [15] and this hormone also induces the accumulation of soluble sugars in *P. patens* [16], the last response was observed in *S. uncinata* revealing a possible control by this hormone.

When *S. uncinata* was submitted to desiccation, experimented a reduction in the metabolic activity, leading to a decrease in respiration and photosynthesis, but cellular respiration was less affected by desiccation at the beginning of the experiment, it has been reported that some plant can maintain their oxygen consumption rate until a 30% of water content, allowing to the plant to maintain a basal energetic level to establish the responses against stress [17, 18]. Photosynthesis was affected rapidly by desiccation, probably because the water is one of the substrates in the photochemical phase; also, the lack of water alters the structure of thylakoid membranes [3].

Photosynthetic efficiency it an indicator of the activity of PSII and the fundament is related to chlorophylls fluorescence when the system is working efficiently the energy is dissipated in the photochemical processes, but when stress is present, the energy is dissipated by non-photochemical phenomena as heat liberation and chlorophyll fluorescence [19]. *S. uncinata* experienced mented a fast decrease in the PSII efficiency, but when the moss was rehydrated, fully recover the activity of PSII, these rapid recovery responses is related to the photosynthetic pigments, because the moss under desiccation no degraded the chlorophylls. The organisms that present this condition are known as poikilochlorophyllic species. The poikilochlorophyllic organisms maintain the chlorophylls and photosystems integrity avoiding the non-enzymatic degradation by photo-oxidative damage [20, 21], using the carotenoids and xanthophylls present in the reaction centers in the photosystems. These compounds protect against an excess of energy using the double conjugated bonds that allow them to capture the excess of energy and dissipate it, process known as non-photochemical quenching [22].

Desiccation affects the fluidity and stability of organelle membranes, allowing the electron leak from the electron chain transport [1], that lead to the generation of reactive oxygen species, like singlet oxygen (^1^O_2_), superoxide ion (O_2_^−^), hydroxyl radical (OH·) and peroxides, mainly the oxygen peroxide [7, 23]. Our results show that *S. uncinata* have an increase in ROS levels, but at the end of the experiment ROS return to basal levels, these are consistent with the grade of lipoperoxidation, the moss experiment a slight increase that was controlled at the 24 h of the desiccation, similar behavior was observed in the moss *F. antipyretica* under fast desiccation treatment [24], but they also show that a slow desiccation allows a better response by the moss, because enables the moss to engage the mechanisms against desiccation minimizing the damage.

In general, the control of the homeostasis redox is due to the action of antioxidant enzymes and non-enzymatic antioxidant like phenolic compounds, tocopherol, glutathione, ascorbate among others [7]. We assay the activity of four antioxidant enzymes, superoxide dismutase (SOD), catalase (CAT), ascorbate peroxidase (APX) and type III peroxidases (POD). These enzymes we capable to control the ROS levels in *S. uncinata* under fast desiccation treatment. SOD acted at the beginning of the desiccation converting the superoxide radical in a less reactive molecule, and then the hydrogen peroxide was detoxified into the water by the action of CAT, APX, and POD where the most active enzyme was POD. *S. uncinata* respond to desiccation not only across the antioxidant responses, also accumulates compatible osmolytes that are highly sensitive to environmental stress [1], this mechanism is not only a preventive response also a tolerance response involves the water shell replacement of molecules inside the cell preventing the protein denaturation and membrane fusion [1, 25]. One of the characteristics of these compounds is that they not interfere with the metabolic activity of the cell and respond to the mechanic stress, loss of turgor and destabilization of membranes [1] also the transcripts related to the sugar machinery increase during dehydration and rehydration [26]. The moss accumulates proline and glycine- betaine, these compounds immobilize the cytoplasm forming a glassy matrix that protect the biomolecules preventing the solvation shell removal, and the decrease in ionic strength [27]. These responses where observed in the moss *Atrichum undulatum* under moderated desiccation. The increasing levels of these osmolytes is due to biosynthesis the *novo*, because not significant increase was observed until the 9 h of desiccation for proline ant 12 h to glycine-betaine, the biosynthesis occurs first because de transcripts of the P*5CS* were induced since the 3rd hour of treatment while the transcript of BADH was induced since the 9th hour of treatment and the osmolyte increase was discrete when is compared to the increase of proline. Proline is a relevant molecule not only for the osmolyte action but also has the capacity to chelate metals, stabilizes molecules like lipids and proteins and acts like an antioxidant and hydrotrope [28, 29]. Glycine-betaine contributes to the osmotic adjustment and stabilizes biomolecules, protecting the membranes of thylakoids and RUBISCO [28, 30]. *S. uncinata* presents high and low molecular weight dehydrins. Dehydrins can interact with all the biomolecules of the cell, like proteins, lipids and nucleic acids, are widely distributed in the cell, including the nucleus, chloroplasts, mitochondrion, and cytoplasm [27]. Dehydrins are part of the primary response against desiccation because of conferee preferential hydration of molecules when the intracellular water potential decrease [1]. Dehydrins also can sequester ions and due to their hydrophilic properties are able to form hydrocolloid that stabilizes the cytoplasm [31]. The moss *B. argenteum* shows a plastic response under desiccation that involves the activation of the transcription and translation machinery, cytoskeleton, sugar metabolism and secondary metabolism [32]. Also, studies of the proteome of the moss *P. patens* under a desiccation treatment shows an increase in group 2 of LEA proteins, dehydrins, and heat shock proteins [33]. A similar response was found in *S. uncinata.* Under control conditions present 13 isoforms (20, 21, 22, 24, 26, 27, 28, 29, 50, 70, 80, 100, 150 kDa) but when is desiccated present 14 isoforms, the 24 kDa isoform is not present and two new forms 25 and 40 kDa accumulates, also *S. uncinata* experiment an increment in the levels of the 19, 21, 50, 70, 80, 100 and 150 kDa dehydrins, showing a dynamic response, and probably the pattern would change during rehydration. The responses of mosses against dehydration are dynamic [32] but they possess some constitutive mechanisms of protection that allow mosses to respond against desiccation despite the rate of water loss and according to our findings *S. uncinata* have the same behavior.

## Conclusion

*Sanionia uncinata* experienced a rapid loss of water, during the drying treatment*. S. uncinata* can tolerate desiccation due to the establishment of mechanisms that involves the control of the homeostasis redox, the osmotic adjustment and the accumulation of molecules like osmolytes and dehydrins that stabilizes the cell and their components, and the responses observed offers new insights in the Antarctic moss responses to water scarcity during climate change.

## Materials and methods

### Plant material

Plants of *S. uncinata* were collected from soil on King George Island, Maritime Antarctic and, the moss was cleaned and rinsed with water, then was cultured with liquid BCD media at 10 ± 2 °C, and 16 h of light and 8 h of darkness for 3 months prior desiccation treatment [34, 35]. Before the desiccation treatment, the moss was rinsed with water and maintain fully hydrated for 30 days. The fully hydrated state was considered as the 100% of relative water content of the moss and 3 cm shoots were placed in culture microplates (12 wells) inside a desiccator with silica gel bags, the silica treatment represents fast desiccation rate; we consider the moss desiccated when the mass was constant in the time, approximately a loss in 95% of original mass. The samples were collected every 3 h for 24 h and stored at − 80 °C before processing.

### Relative water content

Fresh moss tissue was collected every 3 h measured and the weight was determined immediately, then the tissue was dried using an oven at 65 °C for at least 48 h until the weight was constant [36, 37]. The percentage of water was calculated using Eq. (1).

1 $$\frac{{{\text{Initial}}\;{\text{fresh}}\;{\text{weight}} - {\text{final}}\;{\text{fresh}}\;{\text{weight}}}}{{{\text{Initial}}\;{\text{fresh}}\;{\text{weight}}}} \times 100.$$

### Photosynthetic efficiency of photosystem II (PSII)

A photosynthetic efficiency analyzer (FMS II, Hansatech, Norfolk, UK) was used to measure photosynthetic efficiency through the determination of the variable and maximum fluorescence of PSII. The moss was dark adapted for 15 min covering the system with aluminum foil. The optic fiber was placed on the tip of the mosses using an adapter (provided by the manufacturer), ensuring that every measuring was made at the same distance. Results are expressed as F_v_/F_m_ (maximum efficiency of PSII).

### Photosynthetic pigments (Chl-*a*/*b*)

The total content of Chl-*a* and Chl-*b* was determined using fresh tissue (100 mg) that was ground to a powder using liquid nitrogen, mortar and pestle before extraction in ethanol (1 mL). The extract was centrifuged at 10,000 rpm for 10 min at 4 °C and the absorbance of the supernatant at 470, 649 and 665 nm was measured [21]. Chlorophyll concentration was determined using Eqs. (2) and (3). Carotenoids concentration was determined using Eq. (4)

2 $${\text{Chl-}}a\, \left( {\upmu{\text{g}}/{\text{mL}}} \right) = 13.36 \cdot {\text{A}}_{665} {-}5.19 \cdot {\text{A}}_{649}$$

3 $${\text{Chl-}}b\, \left( {\upmu{\text{g}}/{\text{mL}}} \right) = 27.43 \cdot {\text{A}}_{649} {-} \, 8.12 \cdot {\text{A}}_{665}$$

4 $${\text{C}}_{{{\text{x}} + {\text{c}}}} \, \left( {\upmu{\text{g}}/{\text{mL}}} \right) = {{\left( {1000 \cdot {\text{A}}_{470} {-} \, 2.13{\text{ C}}_{\text{a}} - 97.63{\text{ C}}_{\text{b}} } \right)} \mathord{\left/ {\vphantom {{\left( {1000 \cdot {\text{A}}_{470} {-} \, 2.13{\text{ C}}_{\text{a}} - 97.63{\text{ C}}_{\text{b}} } \right)} {209}}} \right. \kern-0pt} {209}}.$$

### Total reactive oxygen species (ROS)

Total ROS was analyzed using fluorometric quantitation of dichlorodihydrofluorescein-diacetate (DCDHF-DA) method. Fresh plant tissue (100 mg) was incubated in 1 mL of 10 µM of DCDHF-DA prepared in Tris–HCl (50 mM, pH 8.0) for 1 h at room temperature. Tissue was then rinsed with EDTA 50 mM to remove the excess of DCDHF-DA, the tissue was ground to a fine powder and extracted in 1 mL of Tris–HCl (50 mM, pH 8.0). The supernatant obtained after centrifugation at 10.000 rpm for 5 min (Heraeus Biofuge fresco, Kendro Laboratory, Hong Kong), fluorescence intensity was measured using a 488 nm wavelength for excitation and 535 nm [38].

### Membrane peroxidation

Fresh tissue (100 mg) was ground to a powder and suspended in 1 mL of 1% of trichloroacetic acid (TCA), then was centrifuged at 10,000 rpm for 5 min. 250 μL of the supernatant was added to 750 μL of 0.5% of thiobarbituric acid in 20% and the mixture was boiled for 15 min, after this procedure the mixture was cooled to room temperature and the adduct formed by TBA-malondialdehyde (MDA) was quantified at 532 nm using ∑ = 155 mM^−1^ cm^−1^ [39].

### Enzymes extraction

Fresh tissue (100 mg) was ground to a fine powder and extracted in 1 mL of potassium phosphate buffer (50 mM, pH 7.5). The mixture was centrifuged at 10,000 rpm for 10 min at 4 °C. The supernatant was recovered, and the concentration of the soluble proteins was determined using Bradford method [40] using bovine serum albumin (BSA) standard curve.

### Superoxide dismutase (SOD) activity (EC 1.15.1.1)

A reaction mixture was prepared using 600 μL of potassium phosphate buffer (50 mM, pH 7.5), 10 μL of 10 mM EDTA, 100 μL of 130 mM methionine, 10 μL of 2 mM riboflavin, 200 μL of 3 mM of nitroblue tetrazolium in 70% dimethylformamide and 100 μL of protein extract. The mixture was incubated under white light for 15 min at room temperature (a blank was kept in the dark). Absorbance was determined at 560 nm (spectrophotometer Espectr BID1, Agilent 8453, Santa Clara, USA) and expressed according to the capacity to inhibit 50% of photochemical reduction of NBT (50% of the photochemical reduction of NBT equals 1EU) [41].

### Ascorbate peroxidase (APX) activity (EC 1.11.1.11)

A reaction mixture that contained 935 μL of potassium phosphate buffer (50 mM, pH 7.5), 20 μL of protein extract, 5 μL of 100 vol. hydrogen peroxide and 40 μL of 10 mM sodium ascorbate were prepared. Absorbance was recorded at 290 nm for 1 min (spectrophotometer Espectr BID1, Agilent 8453, Santa Clara, USA), the activity was expressed according to the ascorbate consumption using molar extinction of ascorbate, ∑ = 2.8 mM^−1^ cm^−1^ [42].

### Peroxidase (POD) activity (EC 1.11.1.7)

A reaction mixture that contained 980 μL of sodium phosphate buffer (50 mM, pH 7.5), 10 μL of protein extract, 5 μL of 100 vol. hydrogen peroxide and 5 μL of guaiacol were prepared. Absorbance was recorded at 470 nm for 1 min (spectrophotometer Espectr BID1, Agilent 8453, Santa Clara, USA). POD activity was expressed in terms of tetrahydroguaiacol (THG) formation using molar extinction of THG, ∑ = 26.6 mM^−1^ cm^−1^ [43].

### Catalase (CAT) activity (EC 1.11.1.6)

A reaction mixture that contained 975 μL of potassium phosphate buffer (50 mM, pH 7.5), 20 μL of protein extract and 5 μL of 100 vol. hydrogen peroxide was prepared. Absorbance was measured at 240 nm for 1 min (spectrophotometer Espectr BID1, Agilent 8453, Santa Clara, USA). CAT activity was expressed in terms of hydrogen peroxide consumption using the molar extinction of hydrogen peroxide, ∑ = 39.4 mM^−1^ cm^−1^ [42].

### ABA content

Fresh tissue (100 mg) was ground to a fine powder with liquid nitrogen and extracted in 1 mL of methanol: formic acid: water 15:4:1. The mixture was centrifuged at 10,000 rpm for 10 min at 4 °C. The supernatant was recovered and filtered (0.22 µm). A volume of 20 µL of the extract was injected in a rheodyne valve into an HPLC–ESI–MS/MS system (Agilent LC–MS/MS 1200s-6410, Agilent Technologies, Santa Clara, CA, USA) equipped with a C18-reversed-phase column (150 × 4.6 mm, 5 µm, XDB-C18, Agilent Technologies, Santa Clara, USA), the mobile phase consists in a solution of 0.1% formic acid, the running was set at a flow rate of 0.3 mL/min at room temperature. The detector was set in MRM mode (multiple reaction monitoring) at − 4500 V, 25 psi, and a 10 mL/min flow rate of nitrogen. ABA (263 → 153 m/z) was used as standard (Sigma-Aldrich, MO, USA) and d6-ABA (269 → 159, Olchemim Ltd., Czech Republic) as an internal standard.

### Proline and glycine betaine content

A volume of 200 µL of enzyme extraction was filtered and 20 µL was injected in a rheodyne valve into an HPLC–ESI–MS/MS system (Agilent LC–MS/MS 1200s-6410, Agilent Technologies, Santa Clara, CA, USA) equipped with an Astec Chirobiotic™ column (150 × 21 mm, 5 µm pore size), the mobile phase consisted of a mixture of 0.1% of formic acid (A) and acetonitrile (B) 95.5:0.5 respectively, the flow rate was 0.5 mL/min. The detector was set in MRM mode (multiple reaction monitoring) at 4000 V, 35 psi, and a 9 mL/min flow rate of nitrogen. Proline (116 → 70 m/z), Gly-betaine (235 → 118 m/z) and were used as standards (Sigma-Aldrich, MO, USA).

### Soluble sugar content

Fresh tissue (100 mg) was ground to a fine powder with liquid nitrogen and extracted in 1 mL of 85% ethanol. The extract was centrifuged at 10,000 rpm for 10 min at 4 °C. The supernatant was recovered and filtered (0.45 µm). A volume of 20 µL of the extract was injected in a rheodyne valve into an HPLC-RID system (Agilent 1100, Agilent Technologies, Santa Clara, CA, USA) equipped with a Sugar Pak I column 300 mm × 6.5 mm (Waters, Waters Corp., Massachusetts, USA), the mobile phase consisted in a preboiled solution of Calcium EDTA 50 mg/L and the running was set to a flow rate of 0.35 mL/min at room temperature. The refractive index detector was set at 55 °C and d-glucose, d-fructose, d-galactose, galactinol, sucrose, raffinose, stachyose and verbascose were used as standards (Sigma-Aldrich, St. Louis, USA).

### Dehydrins Western Blotting

Protein extraction was prepared according to the protocol described above. The supernatant was collected and a volume of cooled acetone (− 20 °C) was added to the sample an incubated for 1 h at − 20 °C, the extract was centrifuged at 10,000 rpm for 10 min at 4 °C. The pellet was resuspended in phosphate buffer (50 Mm, pH 8.0), proteins were standardized by Bradford and a volume of 15 µL was injected into the electrophoresis pocket. A broad range of protein molecular weight was used as a marker. Electrophoresis was performed for 2 h at 120 V. The product was transferred to a nitrocellulose membrane (Merck Millipore Ltda, Tullagreen, USA) using a 300 A for 1 h. The membrane was incubated for 1 h with 5% of fat free milk prepared in TTBS, the membrane was rinsed 3 times with TTBS and were incubated for 1 h at room temperature with the primary antibody anti-DHN 1:1000 prepared in 3% of fat free milk in TTBS (Agrisera, Sweden) o were incubated also for 1 h with the primary antibody anti-RbcL 1:20,000, after the incubation the membrane was rinsed for 15, 10, 5 min with TTBS and the membranes were incubated with the secondary antibody anti-Rabbit IgG HRP conjugated (Agrisera, Sweden) de 1:10,000, after antibody treatment, the membranes were incubated for 3 min with Luminata Forte substrate (Merck, Tullagreen, USA) and the chemiluminescence were detected with x-ray films (Fujifilm).

### Analysis of dhnA, gols, badh, p5cs and rrna18S gene transcript levels

Total RNA was extracted per the manufacturer instructions described in the ISOLATE II RNA plant kit (Bioline reagents Ltda, London, United Kingdom), the purity was determined using the nanoquant assay (Infinite M200pro, Tecan, Sweden). The transcript quantification was assayed using the one step Brilliant II SYBR Green QRT-PCR1 master mix (Agilent Genomics, Santa Clara, USA). The primers set are described in Table 1.

**Table 1:** Primer set and temperature of melting for each gene (Tm)

Gene	Primers set	Tm (°C)
dhnA	AAACTCAGACGCGAGAGTCG TAAGGAGCGGGGAATGTTCG	60
gols	GTCCATTATTGTGCAGCGGG TGGGGTTTGCACACATACAC	60
*P5CS*	CTGCTGTTGTCACTCGGTCT CCAACCCTCGACTTGTCTCC	60
*BADH*	ATGAGCATCCCAATTCCCCA ATCGAGCGGTTTTCCACAGT	60
rrna18S	CTTAGCAGAACGACCAGCGA TCTTCATCGATGCGAGAGCC	60

## Data Availability

All data generated or analyzed during this study are included in this published article.

## Abbreviations

LEA: late embryogenesis proteins
ROS: reactive oxygen species
SOD: superoxide dismutase
APX: ascorbate peroxidase
CAT: catalase
POD: type III peroxidases
ABA: abscisic acid
PSII: photosystem II
Fv: variable fluorescence
Fm: maximum fluorescence
Chl-*a*: chlorophyll *a*
Chl-*b*: chlorophyll *b*
C_x+c_: carotenoids
EDTA: ethylenediaminetetraacetic acid
DCDHF-DA: dichlorodihydrofluorescein-diacetate
TCA: trichloroacetic acid
TBA: thiobarbituric acid
MDA: malondialdehyde
BSA: bovine serum albumin
NBT: nitroblue tetrazolium
THG: tetrahydroguaiacol
MRM: multiple reaction monitoring
